# Validity and Advantages of Three-Dimensional High-Frequency Ultrasound in Dermatological Evaluation

**DOI:** 10.3390/diagnostics15020223

**Published:** 2025-01-19

**Authors:** Misaki Kinoshita-Ise, Taiichiro Ida, Tatsuro Iwasaki, Hideaki Iwazaki, Kazuyuki Yokota, Hoshito Taguchi, Manabu Ohyama

**Affiliations:** 1 Department of Dermatology, Kyorin University Faculty of Medicine, Tokyo 181-8611, Japan; tatsuro-iwasaki@ks.kyorin-u.ac.jp; 2 New Area Business Development Initiative, Advantest Corporation, Saitama 349-1158, Japan; taiichiro.ida@advantest.com (T.I.); hideaki.iwazaki@advantest.com (H.I.); kazuyuki.yokota@advantest.com (K.Y.); hoshito.taguchi@advantest.com (H.T.); 3 Department of Dermatology, Keio University School of Medicine, Tokyo 160-8582, Japan

**Keywords:** three-dimensional, high-frequency ultrasound, dermoscopy, trichoscopy, histopathology, skin appendages, hair diseases, skin tumors, noninvasive, imaging technology

## Abstract

**Background/Objectives:** High-frequency ultrasound (HFUS) has been reported to be useful for the diagnosis of cutaneous diseases; however, its two-dimensional nature limits the value both in quantitative and qualitative evaluation. Three-dimensional (3D) visualization might help overcome the weakness of the currently existing HFUS. **Methods:** 3D-HFUS was newly developed and applied to various skin tumors and inflammatory hair diseases to assess its validity and advantages for dermatological use. **Results:** Three-dimensional images were successfully obtained from skin tumors, including basal cell carcinoma, subungual squamous cell carcinoma, Bowen’s disease, and malignant melanoma, as well as inflammatory hair loss diseases including alopecia areata in different disease phases and lichen planopilaris. Vertical and horizontal images were generated from the original 3D image data and assessed in comparison with histopathological and/or dermoscopic images. By additionally obtaining horizontal data, lateral tumor margins at any depth were visualized in tumors. In inflammatory hair loss diseases, signs potentially associated with disease activity and pathology were detected. In addition, horizontal evaluation helped grasp hair cycle status and hair follicle densities. **Conclusions:** These findings suggested that this novel technology holds promise as a robust noninvasive tool to diagnose and evaluate various cutaneous diseases.

## 1. Introduction

Ultrasound has been utilized in various medical fields. In dermatology, the main use of conventional ultrasound has been to detect or examine massive structures such as tumors, cysts, abscesses, and hematomas [1]. In recent years, high-frequency ultrasound (HFUS), generally referring to the machine with >15 MHz transducers, has been developed, and its usefulness for various skin diseases has been reported [2,3]. Its usage is not limited to tumorous lesions but includes inflammatory diseases such as hidradenitis suppurative, acne, and morphea [2]. Moreover, exceedingly high-frequency ultrasound with a wavelength from 29 to 71 MHz enabled clearer delineation of skin appendages such as hair follicles (HFs), sweat glands, and sebaceous glands [3]. Applying this novel ultrasound technique on the scalp, we recently succeeded in detecting pathological changes in major hair diseases [4]. These new technologies may enhance the accuracy of noninvasive diagnosis of skin diseases; however, some drawbacks remain to be addressed. As the devices provide only two-dimensional (2D) vertical images, an immediate grasp of the whole figure of the target is challenging. For tumors, horizontal boundaries cannot be visualized; thus, ultrasound helps little to determine lateral surgical margins. For hair diseases, hair density, the degree of hair miniaturization, and hair cycle status are important to confirm the diagnosis, evaluate disease severity/activity, and strategize the treatment, but these elements cannot be quantitatively analyzed unless horizontal images are obtained. Due to this limitation, some cases still need to undergo scalp biopsy. Furthermore, to successfully visualize the targets, the angle of the transducer needs to be carefully adjusted; therefore the procedure requires skilled hands. This particularly matters, when anatomically distinctive parts, including scalp and nail, are examined. Taking these hardships into consideration, we hypothesized that the addition of three-dimensional (3D) visualization functionality to the current ultrasonographic technology would benefit practitioners and patients in various clinical scenes.

In this pilot study, we newly developed 3D-HFUS technology, an ultrasonographic machine equipped with an automated sensor enabling the digital construction of 3D images of scanned areas, and applied it to healthy skin and cancerous/inflammatory diseases of different anatomical parts to evaluate its validity and advantages in dermatological evaluation.

## 2. Materials and Methods

### 2.1. 3D-HFUS Device

**Figure 1:**
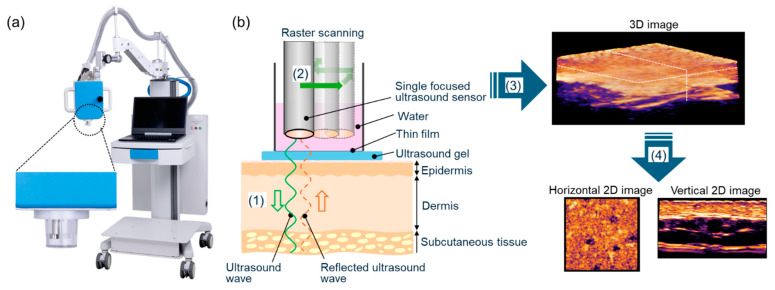
Three-dimensional ultrasound imaging system: (**a**) The appearance of the machine with an enlarged view of the sensor. (**b**) The process of the scanning and the imaging. The focused ultrasound sensor transmits ultrasound wave into the skin and receives the reflected ultrasound wave (1). The sensor scans a 6 × 6 mm area with ultrasound imaging (2). The 3D image is constructed from the captured ultrasound signals (3). The vertical and horizontal 2D images can be generated (4).

The study was performed under the collaboration between Kyorin University and Advantest corporation. All authors were involved in the development of the device. A 3D ultrasound imaging system (Figure 1a) was developed by modifying the previously reported system equipped with 3D photoacoustic and ultrasound imaging (WEL5200, Advantest Corporation, Tokyo, Japan), and this is the first report of its clinical application to in vivo human skin [5].

The validity of this system for the detection of dermal blood vessel structures has already been reported [6]. The laser unit for photoacoustic imaging was removed from the newly developed device to better fine-tune ultrasonographic function for this study. The imaging depth was improved by using a refined ultrasound sensor with a center frequency of 50 MHz and modified F-value. The sensor is installed to automatically move within a resin cylinder with the polyethylenic thin film glued to the bottom. During image data acquisition, purified water is filled in the cylinder and the film is contacted with the object via the coupling gel. Ultrasonic wave is transmitted from the sensor and the reflected wave is received. The received ultrasonic wave is converted into electrical signal in the sensor, and the signal data are transferred to the personal computer system through USB. The data down to the depth of 7 mm was accumulated. The area of 6 × 6 mm is scanned with 15 µm scanning width by a single data collection procedure taking approximately 90 s.

Then, a 3D image was constructed from obtained ultrasonic waveform data. Vertical, horizontal, or sectional images of any angle can be generated by processing the 3D images (Figure 1b).

### 2.2. System Performance Validation

**Figure 2:**
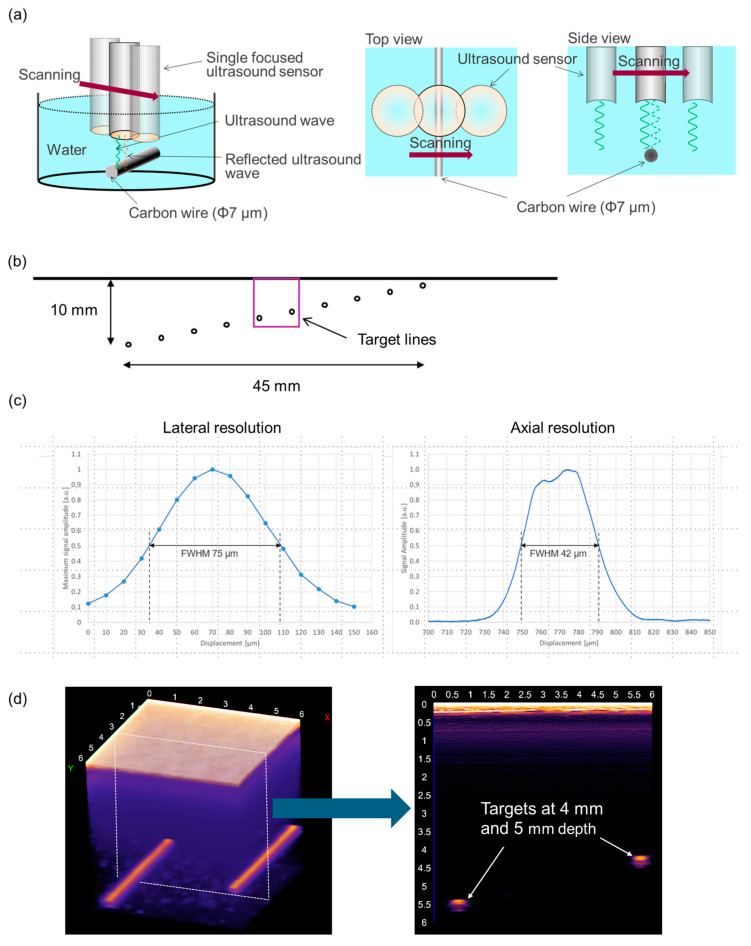
Process and the results of system performance validation: (**a**) The sensor scanned the carbon wire in the container filled with water with 10 µm width steps and the maximum signal amplitude was recorded at each displacement. (**b**) The wires at the depth of 4 mm and 5 mm in the phantom were focused on as targets to visualize. (**c**) The lateral and axial resolutions were calculated by measuring each FWHM. (**d**) The targets at the depth of 4 mm and 5 mm were visualized in 2D and 3D images.

To evaluate the imaging capability of the system, the resolution of the image and the penetration depth were measured. To assess the resolution, the demonstration was performed using a carbon wire with 7 µm diameter (T700SC-12000-50C, Toray Industries, Inc., Tokyo, Japan) in a water-filled container as a phantom. The sensor was mechanically moved to scan the wire with 10 µm scanning width (Figure 2a). The maximum signal amplitude was recorded at each displacement. The Hilbert transform method [7] was applied to plot the ultrasound signal. The lateral (horizontal) and axial (vertical) resolutions were calculated from the FWHM (Full Width at Half Maximum) of the plot [8]. To validate the penetration depth, another phantom (Multipurpose Phantom N-365, Kyoto Kagaku Co., Ltd., Kyoto, Japan) was used. The line targets with a diameter of 100 µm are placed from 1 mm to 10 mm depth with 1 mm spacing in the phantom (Figure 2b). In particular, the detectability of the lines placed at the depth of 4 mm and 5 mm was carefully assessed to examine if this system could depict deep dermis to shallow subcutis.

### 2.3. Scanning of the Healthy Skin

First, to assess how this system depicts healthy skin at different anatomical sites, images were obtained from upper arms (*n* = 10) and palms (*n* = 10) of ten healthy volunteers. The thickness of the epidermis and whole skin (epidermis and dermis) was measured by taking the average of the data from three randomly sampled 2D vertical images that were generated from initial 3D images. The echogenicity (the intensity of reflected echoes) of the dermis in each image was also measured by averaging the brightness of all pixels. Adopting our previous methodology with modifications [4], the brightness was normalized from 0 (the darkest) to 1 (the brightest). The pixels with a brightness under 0.1 were eliminated from the calculation to minimize the influence of the signals derived from skin appendages and blood vessels.

Additionally, images were obtained from the middle fingernail and the scalp of two respective healthy controls to assess how this system depicts structurally distinct anatomical sites of the skin.

### 2.4. Examination of Cutaneous Diseases

After the accumulation of healthy skin data, 3D images of various cutaneous diseases/conditions were collected with corresponding dermoscopic images. The images were, respectively, taken from case 1: nodular basal cell carcinoma (BCC) on the face, case 2: Bowen’s disease on the right leg, case 3: subungual squamous cell carcinoma (SCC) of the left thumb, and case 4: superficial spreading malignant melanoma (MM) on the left neck. In addition, the images were taken from three alopecia areata (AA) patients of different disease phases: case 5: chronic, case 6: acute, and case 7: recovering phases based on clinical and trichoscopic features. Furthermore, the images were taken from case 8: lichen planopilaris (LPP) as a representative of cicatricial alopecia. Horizontal and vertical sectional images were generated from sampled 3D image data. In hair disease cases, the angles of the horizontal sections were adjusted so that the sections and the axis of contained HFs were located perpendicularly. The images were sequentially reviewed by two dermatologists (M.K-I. and T.I. (Tatsuro Iwasaki)) and one technician (T.I. (Taiichiro Ida)).

### 2.5. Comparison Between Histopathological and Matched 3D-HFUS Images

Tumors were excised and subjected to histopathological examination in all skin cancers (cases 1–4). Scalp biopsy was performed in cases 6 and 8; two 4 mm punch samples were taken from a single patient and, respectively, processed into transverse and vertical sections. All samples were stained with Hematoxylin–Eosin. Histopathological and matched ultrasonographic images of the same lesion were retrospectively compared.

## 3. Results

### 3.1. System Performance Validation

Figure 2c shows the result of the lateral (horizontal) and the axial (vertical) resolutions measured using the 7 µm wire. The lateral and axial FWMH were 75 µm and 42 µm, respectively. These values defined the resolution of the system. Figure 2d shows the result of 2D and 3D imaging of the line targets at 4 mm and 5 mm depth. These lines were clearly depicted in the 2D and 3D images, confirming that the system can visualize a tissue at least down to the depth of 5 mm.

### 3.2. Observation of Healthy Skin

**Figure 3:**
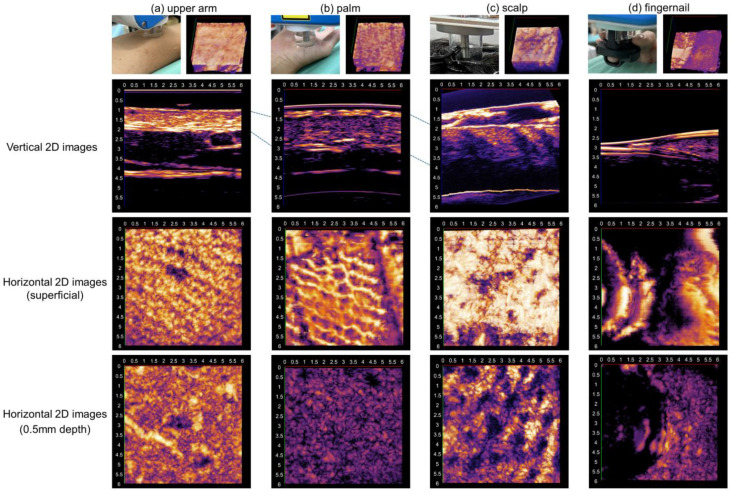
3D-HFUS ultrasonography of healthy skin of different anatomical sites: scanning scenes, 3D original images, and vertical and horizontal 2D images (superficial and 0.5 mm depth) were shown for (**a**) upper arm, (**b**) palm, (**c**) scalp, and (**d**) fingernail.

The system clearly captured the layers of the epidermis, dermis, and subcutis of both upper arms (Figure 3a) and palms (Figure 3b). The epidermis of the arm was detected as a hyperechoic layer and, on the palm, it demonstrated a bilaminar structure, which is compatible with previously reported ultrasonography [2]. In horizontal images, collected from arms and palms, different dermal ridge patterns were observed. Furrows and ridges were more distinctively depicted in the palm (Figure 3a,b). Furrows were visualized as parallelly distributed hyperechoic lines and ridges as the hypoechoic bands alongside furrow lines. Follicular openings were irregularly detected in the upper arms (Figure 3a).

3D-HFUS also depicted distinctive anatomical features of the scalp and nail (Figure 3c,d). In vertical scalp images (Figure 3c), HFs were captured as homogeneous bottom-heavy hypoechoic bands, which is compatible with our previous reports [4]. In horizontal images, which can be obtained by the newly developed system, round to ovoid well-demarcated hypoechoic structures were regularly observed in the dermis. The number of these structures included in the 6 × 6 mm square horizontal image was 22. Based on the following analyses comparing ultrasonographic, trichoscopic, and histopathological data of hair disease cases, these structures were considered to represent follicular units (FUs) which consisted of some HFs mostly in the anagen phase. A fingernail demonstrated a hyperechoic linear line in a vertical section and the epidermis of the nail bed was also hyperechoic and comparable to the echogenicity of the epidermis. The echogenicity of the dermis of the nail bed was lower than that of the nail fold (Figure 3d).

**Figure 4:**
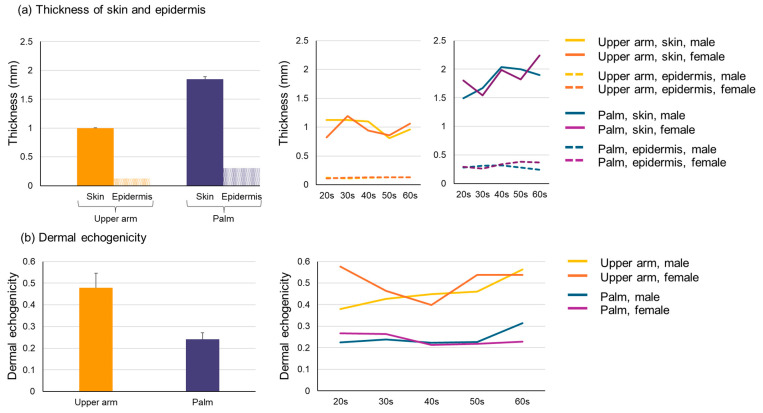
Parameters of skin thickness and dermal echogenicity: (**a**) Thicknesses of the skin and epidermis were calculated for upper arms and palms, respectively. (**b**) The dermal echogenicity was calculated for upper arms and palms, respectively. Average values are shown in the left figures and individual values are plotted in the right figures.

In 3D-HFUS image analysis, the thicknesses of the epidermis and from the epidermis down to the dermis were, respectively, calculated as 0.123 ± 0.008 mm and 0.998 ± 0.131 mm in the flexor aspect of upper arms and 0.307 ± 0.044 mm and 1.849 ± 0.222 in palms (Figure 4a). Both were consistently larger in palms than in upper arms regardless of age and gender, and these values are comparable to previously reported values measured by ultrasound [9,10,11,12]. There was no remarkable difference in the epidermal/skin thickness with age or between females and males (Figure 4a). The dermal echogenicity of the dermis was 0.479 ± 0.067 in the upper arms and 0.242 ± 0.029 in the palms (Figure 4b). The value was consistently higher in upper arms than palms regardless of age and gender (Figure 4b).

### 3.3. Examination of Skin Cancers

**Figure 5:**
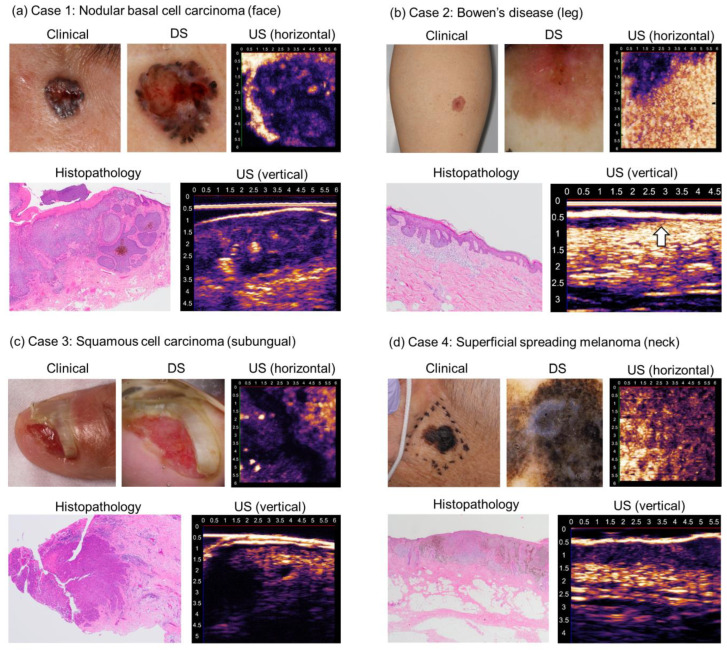
3D-HFUS ultrasonography with clinical, dermoscopic, and histopathological images of skin cancers: 3D-HFUS images (both horizontal and vertical) along with clinical, dermoscopic, and histopathological images are shown for (**a**) nodular basal cell carcinoma on the face, (**b**) Bowen’s disease on the leg (the white arrow indicates the horizontal tumor border), (**c**) Subungual squamous cell carcinoma on the left thumb, and (**d**) Superficial spreading melanoma on the neck. DS: dermoscopy, US: ultrasound.

Skin cancer lesions were depicted more hypoechoically than the surrounding dermis in all tumors. Case 1: nodular BCC (Figure 5a) was captured as a hypoechoic and well-demarcated structure in the dermis, and the gross structure visualized in the vertical image was morphologically comparable to that demonstrated via histopathology. In the horizontal sectional image at the level of the superficial dermis, the tumor boundary was well demarcated and corresponded to that observed by dermoscopy. The tumor contained irregularly hyperechoic areas, which were interpreted as interstitial tissue of the tumor. Case 2: Bowen’s disease (Figure 5b) was depicted as hypoechoic and thickened epidermis, reflecting histopathological changes. The border between the tumor and the underlying dermis was clearly demarcated in the vertical and horizontal images. The horizontal border is indicated by the arrow in Figure 5b. There was no remarkable change in the dermis. Case 3: SCC (Figure 5c) and case 4: MM (Figure 5d) were also depicted as a hypoechoic mass involving the dermis, existing in the epidermis to the shallow dermis. As the 3D-HFUS image was obtained from the center of the lesion in MM, this hypoechoic zone was observed throughout the whole vertical image. The location and the border of the tumors demonstrated in the vertical images were comparable to those via histopathology.

**Figure 6:**
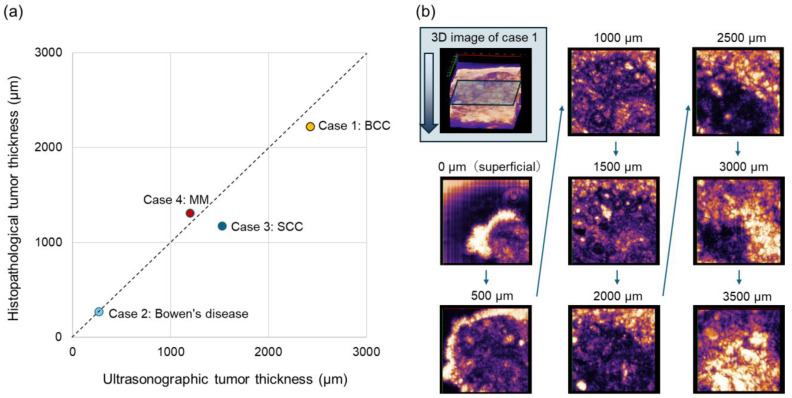
Comparison of histopathological and ultrasonographic tumor thickness and sequential horizontal images: (**a**) Histopathological and ultrasonographic tumor thicknesses of four cancer cases were correlated. BCC: basal cell carcinoma, SCC: squamous cell carcinoma, MM, malignant melanoma. (**b**) Sequential 2D horizontal images of basal cell carcinoma were generated from the original 3D data. Tumor boundaries are followed from the superficial (0 µm depth) to the deepest (3500 µm depth) images.

Ultrasonographic tumor thickness at the deepest part and the corresponding histopathological tumor thickness were well correlated (Figure 6a). Further, horizontal 2D images were sequentially generated from the original 3D image taken from the tumor boundary (case 1). The tumor border in the scanned area was traceable from the most superficial (=0 µm depth) to the deepest dermis (=3500 µm depth), suggesting the usefulness of the invented 3D-HFUS system in surgical margin estimation (Figure 6b).

### 3.4. Examination of Hair Diseases

#### 3.4.1. Alopecia Areata (AA)

**Figure 7:**
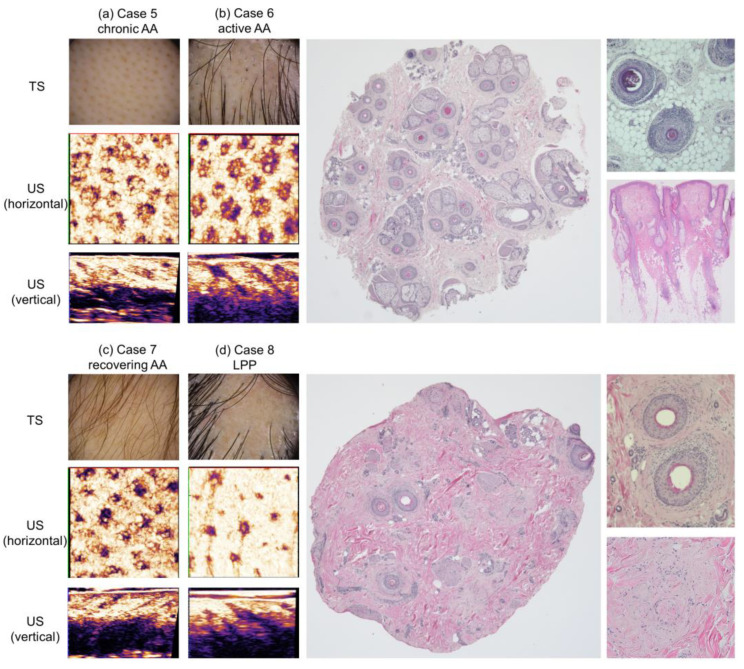
3D-HFUS ultrasonography with trichoscopic and histopathological images of hair diseases: 3D-HFUS images (both horizontal and vertical) along with trichoscopic images are shown for (**a**) chronic alopecia areata (AA), (**b**) active AA, (**c**) recovering AA, and (**d**) LPP (lichen planopilaris). Histopathological images are also shown for (**b**,**d**). TS: trichoscopy, US: ultrasound.

AA manifested different ultrasonographic characteristics depending on the phase of the disease (Figure 7a–c). Similar to the healthy scalp, regularly distributed hypoechoic round to ovoid structures were demonstrated in all horizontal sections. Based on the comparison with histopathological sections of case 6, these structures were identified as FUs. The numbers of FUs included in the 6 × 6 mm square images were comparable to that of the healthy subject: 22 (healthy subject and case 5) and 18 (cases 6 and 7). These numbers well corresponded to those of follicular openings in the same square measures detected by trichoscopy. In case 6, 12 FUs including 28 HFs were detected in the 4 mm punch biopsy horizontal section; these numbers are compatible with those reported as normal HF/FU counts [13,14,15,16]. The density of histopathologically countable FUs (95.5/cm^2^) is higher than that calculated by 3D-HFUS (50.0/cm^2^).

The echogenicity of the inner aspect of these circles was unique and diverse among different disease phases. Hyperechoic signals were homogenously observed in most circles of chronic AA, while the signals were detected as dot core-like in active and recovering AA. Echogenicity in shallow subcutis in active AA was higher than that of chronic and recovering phases corresponding to our previous observation using 2D-HFUS [4]. In the histopathological comparison, most HFs of active AA were identified as those in catagen to early telogen phases, some of which are accompanied by peribulbar inflammation. High echogenicity detected in this area should have reflected these histopathological changes [4]. Ultrasonography of recovering AA (Figure 7c) was comparable with that of a healthy scalp (Figure 3c), but the sizes of the depicted FUs were smaller and their proximal endings were located at the dermis, the position shallower than those of terminal anagen HFs.

#### 3.4.2. Lichen Planopilaris (LPP)

In contrast to AA, the scalp of LPP (Figure 7d) manifested a marked reduction in the number of detectable FUs in horizontal sections reflecting the decreased number of remaining HFs in histopathology: 4 HFs/4 mm punch biopsy sample. The dermis surrounding HFs was homogeneously hyperechoic. In vertical images, the remaining FUs were hardly depicted at the level of the superficial dermis, whereas they were depictable in the deep dermis, in accordance with our previous finding: “distal ambiguity of HFs” [4]. Intriguingly, the remaining FUs presented with distinctive morphological characteristics at the isthmus level, accompanying a linear tract attached to each FU, which resembled the shape of a “tadpole”. In histopathological investigation, these HFs were inflamed and fibrotic at the isthmus level (Figure 7d).

## 4. Discussion

Three-dimensional approaches to visualizing skin tissue using conventional or novel imaging technologies have been attempted in previous studies. These technologies include ultramicroscopy [17], optical coherence tomography (OCT) [18,19], and multiphoton tomography [18]; however, ex vivo skin tissue has been mainly evaluated in these studies, leaving issues to be addressed prior to clinical application. Recently, line-field confocal OCT (LC-OCT), a novel technology with the capability of constructing 3D images, is garnering attention [20]. Some in vivo clinical studies have applied it to various skin diseases, including tumors and inflammatory lesions, and succeeded in clearly visualizing pathological changes. These studies highlighted the potential usefulness of LC-OCT in the dermatology field; however, as the penetration depth is approximately 500 µm [20], its use is limited to the superficial lesions within the epidermis to the shallow dermis, and demands for technology suitable for deeper lesions remain. A few preliminary 3D-HFUS reports examining specific skin tumors/conditions exist [21,22]; however, the usefulness and benefits of 3D-HFUS in clinical settings remain elusive. This pilot study elucidated previously unreported real validity of 3D-HFUS by capturing morphological features and/or signs both in healthy and lesional skin of different anatomical sites and suggested that this novel technology can be widely applied to various clinical situations.

The validation experiments revealed that the lateral and axial resolutions of 3D-HFUS developed in this study are 75 µm and 42 µm, which are comparable to those already reported in 2D-HFUS with quite high-frequency wavelengths: 65 µm (lateral) and 30 µm (axial) [3]. The current system can visualize the skin, at least, down to 5 mm depth. As the majority of dermatological diseases involve the epidermis and dermis, and skin appendages that are embedded within these layers, the visualizing capacity of 3D-HFUS demonstrated in this study supported usability in clinics.

The major advantages of 3D-HFUS include its capability to visualize superficial epidermal ridge patterns and subtle changes in follicular openings in horizontal planes. In cosmetic dermatology, skin texture, wrinkles, and follicular enlargement/plugging are concerns. The influence of aging and skin care procedures/products on the skin can be noninvasively and sequentially monitored by 3D-HFUS. Furthermore, 3D-HFUS successfully depicted the fingernail and scalp, the sites where conventional ultrasound assessment is technically challenging due to their structural complexity. Biopsy of these sites is often avoided or declined because of technical difficulties and post-operational deformities, favoring 3D-HFUS as a novel noninvasive imaging tool for the evaluation of such sites inaccessible by 2D-HFUS.

As demonstrated in sequential horizontal images, the depiction of lateral tumor borders prior to the operation is made possible by 3D-HFUS. This support device is especially helpful when the tumor boundaries cannot be macroscopically or dermoscopically identified or the tumor invades with a bottom-heavy pattern. With pre-operative 3D-HFUS assessment, minimal but substantial excision is more likely to be achieved, which would be particularly beneficial for aesthetically concerned areas including the face. Once standardized, conventional Mohs surgery could be replaced by 3D-HFUS-assisted excision.

3D-HFUS was shown to be useful for the evaluation of inflammatory skin diseases. In this study, we focused on inflammatory hair loss diseases because of the aforementioned major limitations of 2D-HFUS technology. In the preceding study, we successfully depicted pathologically unique and important ultrasonographic signs of alopecia, such as “perifollicular hyperchogenicity in subcutis” in acute AA reflecting peribulbar inflammation, “hypoechogenicity in mid-dermis”, and “distal ambiguity of HFs” in LPP/FFA suggesting the fibrosis and destruction of HF structures [4]. These changes are not directly observable by trichoscopy. In this sense, 2D-HFUS is helpful in diagnosing hair diseases or evaluating their activities/severities; however, the findings remain descriptive and cannot be quantified without obtaining horizontal images containing multiple HFs within the areas of interest. Using 3D-HFUS, horizontal images can be obtained and reviewed. The number of FUs identified by the horizontal review in three AA scalps corresponded well to those of follicular openings detected by trichoscopy. The follicular count by trichoscopy can be challenging on the scalp due to there being many long hairs or conditions such as longstanding AA and androgenetic alopecia, with markedly miniaturized hairs and resultant indetectable follicular openings. As shown in the demonstration of a healthy scalp, 3D-HFUS is minimally influenced by these hurdles, highlighting the advantage of its utility beyond trichoscopy. The number of FUs detected by 3D-HFUS was smaller than those counted on histopathological specimens. This can be explained by the difference in sizes of horizontally detectable FUs in the dermis and superficially detected follicular openings. As the former is much larger than the latter, FUs can be redundantly counted in histopathological specimens. In addition, this discrepancy can be further enhanced by the difference in sample sizes and cutting shapes (6 × 6 mm square vs. 4 mm round).

With the current resolution, FUs containing adjacent HFs were identified but each HF in the same FU was not fully differentiated. Nevertheless, the outcome suggested that cicatricial alopecia is distinguishable at least in its late phase due to the marked decrease in FUs. The size, morphology, and echogenicity of FUs were distinctive among the healthy control, AA of different disease phases, and LPP. Such differences could be attributed to the divergence in hair cycle status, the sizes of HFs and hair shafts, and perifollicular conditions such as inflammation and fibrosis. For example, hyperechoic signals homogeneously observed in most FUs in chronic AA may reflect sebum and keratotic materials in HFs, corresponding to yellow dots in trichoscopy. Unique morphological signs, such as a “tadpole” shape in LPP, are potentially associated with perifollicular fibrosis and inflammation, although further assessment with histopathological comparison is necessary. Obviously, our next aim is to enable thorough quantitative analysis using horizontal data after improving the resolution, which would minimize the chance of unnecessary scalp biopsy. Currently, two lesional 4 mm punch biopsies (for vertical and horizontal sections, respectively) are recommended to fully assess the pathophysiology of hair loss diseases [23]. 3D-HFUS noninvasively and less laboriously facilitates such evaluation, leading to relief not only for patients but also for practitioners. Once a 3D image is taken, any 2D images at different angles can be immediately generated without adjusting the angle of the transducer, highlighting additional advantages of 3D-HFUS. Indeed, the operation time of 90 s per procedure is realistic and manageable in daily clinics.

There are some limitations to be addressed before 3D-HFUS can be authentically used for clinical application. First of all, this is a pilot study with a limited number of cases; thus, the accumulation of data from different skin diseases and additional statistical analysis are necessary. Comparison with other imaging devices (e.g., LC-OCT) would further highlight the advantages and weaknesses of 3D-HFUS, elucidating how to differentiate and combine these new technologies. Three-dimensional findings need to be carefully interpreted as the previous study on 2D-HFUS tackled it by elaborate comparison between histopathology and ultrasonography [24]. As the sensor needs to be placed in a cap filled with water, the application of this technology is challenging for some anatomical sites where the probe capsule needs to be diagonally placed. Physical movement, particularly breathing, can affect the resolution of the images. Currently, the size of the detection window is limited to a 6 × 6 mm square area; thus, multiple scans are required to fully visualize widespread lesions. Because of the nature of high-frequency ultrasound waves, HFUS is suitable for the visualization of sharrow skin. Some modifications, for instance, a combination of multiple ultrasound devices with different wavelengths, are necessary for the assessment of massive targets.

To comprehensively demonstrate the usefulness of 3D-HFUS in clinics, skin tumors and hair diseases were predominantly assessed in this study; however, the application can be expanded to other inflammatory skin diseases. As relatively shallow lesions would be idealistic targets for further validation, diseases with epidermal or interface changes, such as atopic and other dermatitis, blistering diseases, psoriasis, lichen planus, drug eruption, and collagen diseases represent conditions to be assessed in future studies.

In conclusion, a novel HFUS system enabling 3D depiction of normal and diseased skin was successfully invented, which potentially offers clinicians a next-generation solution for current hurdles in the diagnosis/assessment of dermatological problems requiring multiple angle/plane examinations. Room for improvement exists; however, 3D-HFUS has the potential to reduce the necessity of invasive skin biopsy by providing more informative and processable microanatomical images.

## Data Availability

The original contributions presented in this study are included in the article. Further inquiries can be directed to the corresponding authors.
